# COVID‐19‐Related Hematological Abnormalities Among Adults; A Cross‐Sectional Study in a Resource‐Limited Setting

**DOI:** 10.1002/iid3.70322

**Published:** 2026-01-12

**Authors:** Charles Nkansah, Felix Osei‐Boakye, Samuel K. Appiah

**Affiliations:** ^1^ Department of Hematology, School of Allied Health Sciences University for Development Studies Tamale Ghana; ^2^ Department of Medical Laboratory Technology Faculty of Applied Science and Technology, Sunyani Technical University Sunyani Ghana

**Keywords:** anemia, blood cell abnormalities, COVID‐19, hematological parameters, SARS‐CoV‐2, thrombocytopenia

## Abstract

**Background:**

Alterations in hematological parameters in SARS‐CoV‐2 infection may contribute to disease severity and poor outcomes. This study reports the hematological profile of COVID‐19 patients.

**Methods:**

This was a cross‐sectional study involving 169 confirmed COVID‐19 patients conducted at Sunyani Teaching Hospital between January and August, 2022. Sociodemographic, clinical, and laboratory data were obtained. Full blood count was performed using an automated hematology analyzer, and systemic inflammatory indices were calculated.

**Results:**

Participants were mostly young adults (51.5%), females (53.8%), resided in urban settings (41.4%), and aged 20‐81 years with a median age of 35.0 (29.0–47.0) years. Overall, anemia was present in 60.4% of the COVID‐19 patients (56.4% in males and 63.7% in females), with 23.7%, 23.7%, and 13.0% experiencing mild, moderate, and severe anemia, respectively. The COVID‐19 patients mostly had normocytic normochromic anemia (50.3%), and 37.9% had macrocytic normochromic anemia. Older adults (6.600 times, *p* = 0.004), middle‐aged adults (4.435 times, *p* = 0.034), and rural dwellers (3.759 times, *p* = 0.012) were associated with anemia among the COVID‐19 patients. Thrombocytopenia was detected in 56.2% of the patients, and 36.7%, 16.0%, and 2.4% had mild, moderate and severe thrombocytopenia respectively. Leucocyte alterations among the patients included leucopenia (22.5%), leucocytosis (17.2%), neutropenia (40.8%), and lymphocytosis (39.6%). Total leucocyte count (AUC: 0.818, *p* < 0.001), absolute neutrophil count (AUC: 0.816, *p* < 0.001), and absolute lymphocyte count (AUC: 0.804, *p* < 0.001) predicted severe COVID‐19 among the participants. Bicytopenia and pancytopenia were observed in 37.3% and 17.8%, respectively.

**Conclusion:**

COVID‐19 is associated with diverse and clinically relevant hematological abnormalities, remarkably anemia, thrombocytopenia, and leucocyte alterations, and usually relate with disease severity. Routine blood cell analysis is essential in the management of COVID‐19, particularly in resource‐limited settings.

## 1 Introduction

The advent of Coronavirus disease 2019 (COVID‐19), caused by severe acute respiratory syndrome coronavirus 2 (SARS‐CoV‐2), has posed a devastating threat to global health claiming numerous lives and negatively influencing economic stabilities especially in underserved countries [1]. Following the first detection of the beta‐coronavirus in Wuhan, China, in late 2019, approximately 800 million individuals have been infected with over seven million mortalities reported globally. In Africa, the magnitude of COVID‐19 has been substantial, where almost 13 million cases and 258,892 SARS‐CoV‐2‐related deaths have occurred. Since Ghana detected its first two cases of COVID‐19 on March 12, 2020 in samples from two immigrants who had lately returned from Turkey and Norway, the country has suffered approximately 200,000 cases and 1462 deaths [2, 3]. SARS‐CoV‐2 is principally transmitted via respiratory droplets and aerosols during close proximity with infected people, and this promotes the virus' interactions with its primary receptor angiotensin‐converting enzyme‐2 (ACE‐2) especially in the pulmonary region. Owing to the general distribution of ACE‐2 in the body, SARS‐CoV‐2 eventually affects other parts triggering both pulmonary and systemic problems [4]. COVID‐19 presents with a broad spectrum of clinical manifestations, from subclinical cases to severe respiratory failure and multi‐organ dysfunction. Although the respiratory tract is the prime target, COVID‐19 also causes systemic inflammation that induces a massive cytokine storm and eventually affects multiple organs, including the cardiovascular, gastrointestinal, renal, and hemopoietic systems [5].

A notable systemic manifestation of COVID‐19 is the modification of hematological parameters. The direct interaction of SARS‐CoV‐2 with the bone marrow suppresses its hemopoietic activities, and this affects the production, morphology, and function of blood cells, and such hematological disorders are linked with disease progression and severity [5, 6]. Anemia remains one of the most popular hematological abnormalities observed in COVID‐19 patients. Complex mechanisms such as inflammation‐induced suppression of erythropoiesis, direct interaction of SARS‐CoV‐2 with hemopoietic stem cells, dysregulated iron metabolism, haemolysis, and the implication of cytokine storms that retard red blood cell survival and production, are associated with the development of anemia in COVID‐19 [7, 8, 9]. The significant association between anemia and adverse outcomes in SARS‐CoV‐2 infection, including augmented hospitalization, oxygen requirement, and mortality is reported in both low‐ and high‐income countries [10]. In Ghana, where anemia is endemic and still a major “enemy” to public health due to factors such as nutritional deficiencies, parasitic, and chronic conditions, COVID‐19 may aggravate anemia burden among infected persons [6, 11].

Exposure to SARS‐CoV‐2 disrupts immune‐regulatory markers which predispose patients to coagulopathies and other severe complications. Among COVID‐19 patients, thrombocytopenia has been commonly reported, and this may arise from bone marrow suppression, amplified platelet consumption from disseminated intravascular coagulation, and immunologically‐driven platelet lysis. The SARS‐CoV‐2‐induced thrombocytopenia has been linked with poor prognosis and is prominently observed in critically ill COVID‐19 patients [12, 13, 14, 15]. Likewise, leucocyte abnormalities are generally reported in COVID‐19 and this indicates the dynamic immune response to the SARS‐CoV‐2. The initial phase of COVID‐19 may present with leucopenia or lymphopenia, while severe illness mostly results in leucocytosis, neutrophilia, and monocytosis, influenced by systemic inflammation [12, 16, 17, 18].

In addition, immune‐inflammatory ratios such as neutrophil‐to‐lymphocyte ratio (NLR), platelet‐to‐lymphocyte ratio (PLR), platelet‐to‐neutrophil ratio (PNR), and the systemic immune‐inflammation index (SII) have been recognized as surrogate markers for disease severity in COVID‐19. These indices, obtained from basic full blood count parameters, are easier to estimate and show insights into the equilibrium between inflammation and immune proficiency. The unique association between high inflammatory indices with enhanced risk of severe COVID‐19 and death have been recognized. The utility of the inflammatory indices in SARS‐CoV‐2 lies in their ability to recognize high‐risk patients quickly, even before the appearance of explicit clinical deterioration [19, 20, 21, 22, 23].

Regardless of the detected COVID‐19‐induced hematological abnormalities globally, there are few studies from sub‐Saharan Africa, particularly Ghana. Most studies on COVID‐19 in Ghana have prioritized epidemiology, vaccine uptake, and clinical presentations, with limited attention to laboratory and hematological parameters [3, 24]. Given the endemicity of other infections that autonomously alter hematological indices, such as malaria and HIV [6, 11], it is imperative to describe the exclusive hematological profile of COVID‐19 in this setting. Hence, this study assessed the hematological profile of SARS‐CoV‐2‐infected adults. Exploring these alterations is central for guiding suitable interventions, especially in under‐resourced setting where cutting‐edge diagnostic and monitoring techniques may not be readily accessible.

## 2 Materials and Methods

### 2.1 Study Participants

One hundred and sixty‐nine reverse transcriptase‐polymerase chain reaction (RT‐PCR)‐confirmed COVID‐19 adults were recruited for this cross‐sectional study at Sunyani Teaching Hospital between January and August, 2022. The teaching hospital is the main referral and treatment facility situated in the Bono Region, Ghana, and designated as a laboratory confirmation site and COVID‐19 case management center in the region. Sunyani is the administrative capital of the Bono Region and the municipality houses over 100,000 population [25]. The appropriate sample size was estimated using Cochran's formula at a 95% confidence level, 0.05 margin of error, and 13.2% prevalence of COVID‐19 in Ghana [24]. Patients with identified chronic hematological disorders, malignancies, chronic kidney or liver disease, and acute infections unrelated to COVID‐19 were excluded to lessen confounding effects on hematological parameters. Additionally, persons with incomplete clinical records were not enrolled. Participants' socio‐demographics (age, sex and place of residence), vital signs (temperature, blood pressure, pulse and oxygen saturation [SpO_2_]), and other clinical manifestations were retrieved from the medical records.

### 2.2 Sampling and Laboratory Assays

Nasopharyngeal swabs were obtained and mixed with virus transport medium for the diagnosis of COVID‐19 using RT‐PCR technique. The swabs were taken before initiating COVID‐19 medication. The clinical severity was described using the standards categorized by the World Health Organization. Mild/moderate COVID‐19 participants had no or lower evidence of pneumonia or hypoxia, while severe patients experienced respiratory frequency with SpO_2_ < 90% on room air or requires supplemental oxygen [26]. Three milliliters of blood was collected from the COVID‐19 patients for full blood count (Mindray BC3000 Plus, China). The following operational definitions of hematological abnormalities were considered [27, 28]:

- Anemia: Hb < 13.0 g/dL in males (Mild anemia: 11.0–12.9 g/dL, Moderate anemia: 8.0–10.9 g/dL, Severe anemia: < 8.0 g/dL); and < 12.0 g/dL in females (Mild anemia: 11.0–11.9 g/dL, Moderate anemia: 8.0–10.9 g/dL, Severe anemia: < 8.0 g/dL)
- Macrocytosis: MCV > 100 fL
- Microcytosis: MCV < 80 fL
- Hypochromasia: MCH < 27.0 pg
- Thrombocytopenia: Platelet count < 150 × 10⁹/L (Mild thrombocytopenia: 101–149 × 10^9^/L, Moderate thrombocytopenia: 51–100 × 10^9^/L, Severe thrombocytopenia: ≤ 50 × 10^9^/L)
- Leucopenia: WBC count < 4.0 × 10⁹/L
- Leucocytosis: WBC count > 11.0 × 10^9^/L
- Neutropenia: Absolute neutrophil count < 2.0 × 10⁹/L
- Lymphocytosis: Absolute lymphocyte count > 4.0 × 10⁹/L
- Bicytopenia: Reduction in any two of the three cell lines (RBC, WBC, Platelets)
- Pancytopenia: Reduction in all three cell lines
- Morphological forms of anemia were described using standard MCV and MCH cut‐offs:

◦ Microcytic hypochromic: MCV < 80 fL and MCH < 27 pg
◦ Normocytic normochromic: MCV 80–100 fL and MCH 27–33 pg
◦ Macrocytic normochromic: MCV > 100 fL and MCH 27–33 pg

Both the COVID‐19 confirmation and full blood count tests were performed at the Clinical Laboratory of Sunyani Teaching Hospital.

### 2.3 Statistical Analysis

SPSS v.27.0 (Armonk, USA) was used for data analysis. Numerical data were expressed as mean ± SD or median (25^th^–75^th^ percentiles) and comparisons were made using independent samples *T*‐test or Mann–Whitney *U*‐test. Nominal variables were expressed as proportions and compared using *χ* ^2^ test or Fisher's exact test. The receiver operating characteristic (ROC) was performed to evaluate the predictive performance of systemic inflammatory markers for severe COVID‐19. Statistical significance was considered when *p* < 0.05.

### 2.4 Ethics and Informed Consent

All protocols were in line with the Declaration of Helsinki. Ethical clearance was obtained from the Committee on Human Research, Publication and Ethics of Kwame Nkrumah University of Science and Technology, Kumasi, (CHRPE/AP/012/22). Participants provided written informed consent to be part of this study.

## 3 Results

### 3.1 Age, Sex and Residence of the Participants

This study comprised 169 SARS‐CoV‐2‐infected adults aged 20–81 years with a median age of 35.0 (29.0–47.0) years. About 54% were females, and participants were mostly young adults (51.5%), and resided in an urban setting (41.4%) (Table 1).

**Table 1:** Age, sex and residence of the participants.

Variables	Frequency	Percentage (%)
Age (years)		
Median (25^th^–75^th^ percentiles)	35.0 (29.0–47.0)	
Adolescents (≤ 24)	21	12.4
Young adults (25–40)	87	51.5
Middle‐aged adults (41–60)	40	23.7
Old adults (> 60)	21	12.4
Sex		
Males	78	46.2
Females	91	53.8
Residence		
Urban	70	41.4
Peri‐urban	49	29.0
Rural	50	29.6

### 3.2 Clinical Manifestations of the Participants

The clinical manifestations experienced by the SARS‐CoV‐2 patients included headache (39.6%), cough (61.5%), nausea (26.0%), loss of smell (56.2%), loss of taste (57.4%), fatigue (51.5%), and fever (59.8%). Almost one‐third of the 269 participants had severe COVID‐19 and required immediate care. Averagely, the participants had moderately increased temperature (37.6°C ± 1.3°C), systolic blood pressure (132.0 [123.0–147.0] mmHg) and diastolic blood pressure (81.0 [72.0–88.0] mmHg). While participants' SpO2 was at a borderline (94.0 [90.0–97.0] %), their pulse rate was normal 90.0 [84.0–97.0] bpm) (Table 2).

**Table 2:** Clinical manifestations of the participants.

Variables	Frequency	Percentage (%)
Signs and symptoms		
Headache		
Present	67	39.6
Absent	102	60.4
Cough		
Present	104	61.5
Absent	65	38.5
Nausea		
Present	44	26.0
Absent	125	74.0
Loss of smell		
Present	95	56.2
Absent	74	43.8
Loss of taste		
Present	97	57.4
Absent	72	42.6
Fatigue		
Present	87	51.5
Absent	82	48.5
Fever		
Present	101	59.8
Absent	68	40.2
Disease severity		
Severe COVID‐19	53	31.4
Mild/Moderate COVID‐19	116	68.6
Vital signs		
Temperature (°C)	37.6 ± 1.3	
Systolic BP (mmHg)	132.0 (123.0–147.0)	
Diastolic BP (mmHg)	81.0 (72.0–88.0)	
SpO_2_ (%)	94.0 (90.0–97.0)	
Pulse (bpm)	90.0 (84.0–97.0)	

### 3.3 Blood Cell Parameters of COVID‐19 Patients

Overall, the COVID‐19 adults were mildly anemic (Hb = 11.1 ± 2.4 g/dL) and thrombocytopenic (platelet count = 132.0 [105.4–182.9] × $10^{9}$/L), but leucocyte count was within the normal range. Red blood cells, Hb, and HCT were lower in SARS‐CoV‐2‐infected females than their male counterparts. Platelet and leucocyte parameters, and systemic inflammatory ratios (NLR, PLR, PNR, and SII) were comparable between male and female COVID‐19 patients (Table 3).

**Table 3:** Blood cell parameters of COVID‐19 patients.

Blood cell parameters	COVID‐19 Patients			*p* value
	Total	Males (*n* = 78)	Females (*n* = 91)	
RBC ($\times 10^{12}$/L)	3.5 ± 0.8	3.7 ± 0.8	3.3 ± 0.8	< 0.001
Hb (g/dL)	11.1 ± 2.4	11.6 ± 2.4	10.6 ± 2.2	0.004
HCT (%)	33.0 ± 7.3	35.7 ± 7.3	30.7 ± 6.4	< 0.001
MCV (fL)	95.7 ± 11.7	95.1 ± 13.2	96.3 ± 10.3	0.526
MCH (pg)	33.3 ± 4.9	32.8 ± 5.0	33.7 ± 4.8	0.267
MCHC (g/dL)	34.6 ± 2.0	34.3 ± 1.4	35.0 ± 2.4	0.026
RDW‐CV (%)	14.6 (13.6–15.5)	14.5 (13.6–15.0)	14.8 (13.5–15.8)	0.133
WBC (×$10^{9}$/L)	6.1 (4.2–9.7)	5.3 (3.9–9.7)	6.4 (5.4–9.7)	0.062
Neutrophil (×$10^{9}$/L)	2.1 (1.6–2.9)	2.0 (1.4–2.7)	2.1 (1.8–3.1)	0.058
Lymphocyte (×$10^{9}$/L)	3.3 (2.1–6.2)	3.0 (1.9–6.5)	3.6 (2.8–6.2)	0.184
MID (×$10^{9}$/L)	0.4 (0.3–0.6)	0.4 (0.3–0.6)	0.4 (0.3–0.7)	0.210
Platelet (×$10^{9}$/L)	132.0 (105.4–182.9)	132.0 (108.0–179.8)	135.0 (103.0–212.0)	0.514
MPV (fL)	8.3 (7.6–8.8)	8.3 (7.7–9.4)	8.4 (7.5–8.8)	0.375
PDW (%)	15.4 (11.7–15.9)	15.4 (11.9–15.9)	15.3 (7.1–15.8)	0.085
NLR	0.6 (0.4–0.8)	0.5 (0.4–0.8)	0.6 (0.5–0.8)	0.668
PLR	53.1 (17.3–75.4)	52.9 (17.3–75.4)	53.1 (16.3–77.5)	0.707
PNR	66.3 (44.1–121.2)	65.3 (46.7–111.0)	69.6 (41.2–121.2)	0.635
SII	91.0 (45.9–142.1)	80.5 (49.6)	92.4 (45.9–150.4)	0.201

### 3.4 Age, Vital Signs and Blood Cell Parameters Based on COVID‐19 Severity

The severe COVID‐19 adults were relatively older than those in the mild/moderate group. With the exception of diastolic blood pressure which was similar between the two groups, adults with severe COVID‐19 had elevated axillary temperature, SBP, and pulse rate than the mild/moderate COVID‐19 adults. On the contrary, severe COVID‐19 patients had lower SpO_2_ (*p* < 0.001) compared to the mild/moderate adults.

RBC count, hemoglobin, hematocrit, and platelet count were lower in the severe than mild/moderate COVID‐19 patients. Adults with severe COVID‐19 had lower red cell indices (MCV and MCH), but higher red cell distribution‐width than the mild/moderate group. Leucocyte parameters: white blood cell count, absolute neutrophil count, absolute lymphocyte count, and MID (monocytes, eosinophils and basophils) were elevated in patients with severe COVID‐19 than those with mild/moderate disease. Also, systemic inflammatory ratios: NLR, PLR, PNR, and SII were elevated among the severely ill adults than the mildly/moderately ill COVID‐19 patients (Table 4).

**Table 4:** Age, vital signs and blood cell parameters based on COVID‐19 severity.

Variables	Mild/moderate COVID‐19 (*n* = 116)	Severe COVID‐19 (*n* = 53)	*p* value
Age (years)	33 (28.0–43.50)	44 (31.5–55.5)	< 0.001
Vital signs			
Temperature (°C)	37.3 ± 1.5	38.3 ± 0.6	< 0.001
Systolic BP (mmHg)	130.0 (116.5–142.0)	153.0 (128.0–159.0)	< 0.001
Diastolic BP (mmHg)	79.0 (72.0–86.0)	84.0 (74.5–91.0)	0.117
SpO_2_ (%)	95.0 (92.0–97.0)	88.0 (87.0–94.0)	< 0.001
Pulse (bpm)	86.5 (83.0–94.0)	97.0 (87.5–104.5)	< 0.001
Full blood count			
RBC ($\times 10^{12}$/L)	3.7 ± 0.8	2.9 ± 0.7	< 0.001
Hb (g/dL)	12.3 ± 1.5	8.5 ± 1.8	< 0.001
HCT (%)	36.0 ± 5.6	26.4 ± 6.0	< 0.001
MCV (fL)	97.6 ± 11.2	91.6 ± 11.9	0.002
MCH (pg)	33.8 ± 4.7	32.1 ± 5.2	0.039
MCHC (g/dL)	34.5 ± 1.8	34.9 ± 2.5	0.351
RDW‐CV (%)	14.4 (13.2–15.1)	14.9 (14.5–16.4)	< 0.001
WBC (×$10^{9}$/L)	5.3 (3.7–6.8)	9.9 (8.3–13.1)	< 0.001
Neutrophil (×$10^{9}$/L)	1.9 (1.2–2.4)	3.0 (2.6–3.9)	< 0.001
Lymphocyte (×$10^{9}$/L)	2.8 (1.9–3.8)	6.5 (5.1–9.3)	< 0.001
MID (×$10^{9}$/L)	0.4 (0.3–0.6)	0.6 (0.3–0.8)	0.024
Platelet (×$10^{9}$/L)	162.5 (117.4–210.3)	91.8 (76.3–116.0)	< 0.001
MPV (fL)	8.3 (7.4–9.1)	8.3 (8.2–8.5)	0.962
PDW (%)	15.4 (12.0–15.9)	15.5 (8.7–15.9)	0.392
NLR	0.5 (0.3–0.7)	0.6 (0.5–0.8)	< 0.001
PLR	14.1 (7.9–36.3)	58.4 (49.3–83.5)	< 0.001
PNR	32.0 (22.3–48.1)	95.7 (59.7–145.3)	< 0.001
SII	38.3 (30.6–67.9)	108 (80.5–152.0)	< 0.001

### 3.5 Blood Cell Abnormalities of COVID‐19 Patients

Anemia was present in about two‐third of the COVID‐19 patients, and mild, moderate and severe anemia occurred among 40 (23.7%), 40 (23.7%), and 22 (13.0%), respectively among the participants. The prevalence of anemia in males was 56.4%, whereas 63.7% of the female participants were anemic. In males, 24.4%, 24.4%, and 7.7% had mild, moderate and severe anemia, and the proportion of females with mild, moderate and severe were 23.1%, 23.1%, and 17.6%, respectively. Other erythrocyte abnormalities identified among COVID‐19 adults were macrocytosis (37.9%), microcytosis (7.7%), and hypochromasia (7.7%). Red blood cell distribution width was high in 52.1%, but decreased in 11.2% of the participants. With regards to the forms of anemia, normocytic normochromic anemia was the most dominant (50.3%) found among the COVID‐19 patients, followed by macrocytic normochromic anemia (37.9%). Only few of the COVID‐19 patients had microcytic normochromic (4.1%), normochromic hypochromic (4.1%), and microcytic hypochromic anemia (3.6%). About 60% of the COVID‐19 patients had normal white blood cell count, while 22.5% and 17.2% presented with leucopenia and leucocytosis, respectively. Neutropenia was present in 40.8% of the participants, and 39.6% had lymphocytosis. Moreover, 56.2% of the COVID‐19 patients were thrombocytopenic, and the proportion of participants with mild, moderate and severe thrombocytopenia found were 36.7%, 16.0%, and 2.4%, respectively. Platelet microcytosis was seen in about one‐third of the COVID‐19 adults, and 14.8% had low platelet distribution width. About one‐fifth of the participants had normal blood cells, and single cell abnormality, bicytopenia and pancytopenia was observed in 27.2%, 37.3%, and 17.8%, respectively (Table 5).

**Table 5:** Blood cell abnormalities of COVID‐19 patients.

Variables	Comments		Reference interval	Frequency	Percentages
Hemoglobin (g/dL)	Males	Normal	≥ 13.0	34	43.6
		Mild anemia	11.0–12.9	19	24.4
		Moderate anemia	8.0–10.9	19	24.4
		Severe anemia	< 8.0	6	7.7
	Females	Normal	≥ 12.0	33	36.3
		Mild anemia	11.0–11.9	21	23.1
		Moderate anemia	8.0–10.9	21	23.1
		Severe anemia	< 8.0	16	17.6
MCV (fL)	Normal		80.0–100.0	92	54.4
	Microcytosis		< 80.0	13	7.7
	Macrocytosis		> 100.0	64	37.9
MCH (pg)	Normal		27.0–31.0	156	92.3
	Hypochromasia		< 27.0	13	7.7
RDW‐CV (%)	Normal		11.0–14.5	62	36.7
	Low		< 11.0	19	11.2
	High		> 14.5	88	52.1
Leucocytes (×10^9^/L)	Normal		4.0–11.0	102	60.4
	Leucopenia		< 4.0	38	22.5
	Leucocytosis		> 11.0	29	17.2
Neutrophils (×$10^{9}$/L)	Normal		2.0–7.5	100	59.2
	Neutropenia		< 2.0	69	40.8
Lymphocytes (×$10^{9}$/L)	Normal		1.0–4.0	102	60.4
	Lymphocytosis		> 4.0	67	39.6
Platelet (×$10^{9}$/L)	Normal		150–450	74	43.8
	Mild thrombocytopenia		101–149	62	36.7
	Moderate thrombocytopenia		51–100	27	16.0
	Severe thrombocytopenia		≤ 50	4	2.4
MPV (fL)	Normal		8.0–12.5	114	67.5
	Low		< 8.0	55	32.5
PDW (%)	Normal		10.0–17.9	141	83.4
	Low		< 10.0	25	14.8
	High		> 17.9	3	1.8
Multiple abnormalities	No abnormality		—	30	17.8
	Single abnormality		—	46	27.2
	Bicytopenia		—	63	37.3
	Pancytopenia		—	30	17.8

### 3.6 Relationship Between Socio‐Demographics and Anemia Severity Among the COVID‐19 Patients

Severe anemia was more common among the older (68.8%) and middle‐aged (30.8%) adults than the adolescents and the young adults with COVID‐19. There was an association between anemia severity, and participants' ages and residence. Older adults with COVID‐19 were 6.600 times (*p* = 0.004), and middle‐aged adults were 4.435 times (*p* = 0.034) more likely to experience severe anemia than the adolescents. Again, the chances of participants who resided in rural settings were 3.759 times (*p* = 0.012) higher than those living in the urban areas in getting anemia (Table 6).

**Table 6:** Relationship between socio‐demographics and anemia severity among the COVID‐19 patients.

Variables	Anemic COVID‐19 patients				
	Mild/moderate anemia (*n* = 80, 47.3%)	Severe anemia (*n* = 22, 13.0%)	*p* value	aOR (95% CI)	*p* value
Age group			< 0.001		
Adolescents	9 (100)	0 (0)		1	—
Young adults	48 (94.1)	3 (5.9)		2.031	0.291
Middle‐aged adults	18 (69.2)	8 (30.8)		4.435	0.034
Old adults	5 (31.3)	11 (68.8)		6.600	0.004
Sex			0.09		
Males	38 (86.4)	6 (13.6)		—	—
Females	42 (72.4)	16 (27.6)		—	—
Residence			< 0.001		
Urban	39 (95.1)	2 (4.9)		1	—
Peri‐urban	26 (100)	0 (0)		1.888	0.189
Rural	15 (42.9)	20 (57.1)		3.759	0.012

### 3.7 ROC Analysis to Predict the Diagnostic Performance of Immune‐Inflammatory Markers for Severe COVID‐19

**Figure 1:**
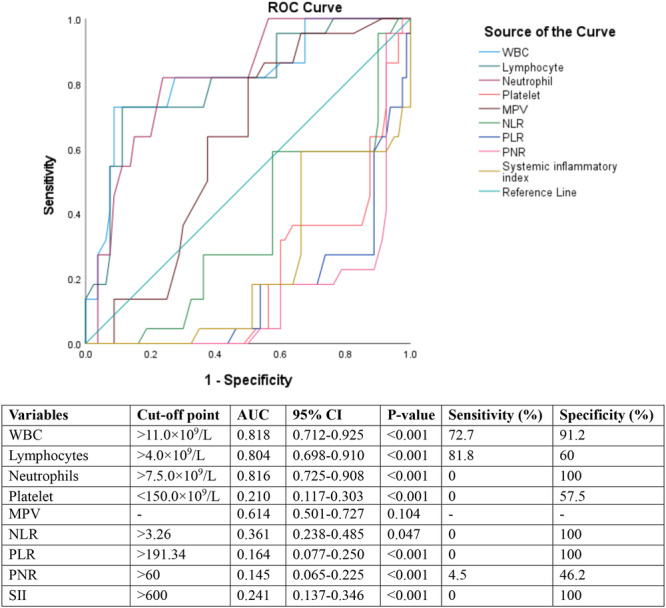
ROC analysis to predict the diagnostic performance of immune‐inflammatory markers for severe COVID‐19.

Total leucocyte count (AUC: 0.818, *p* < 0.001), absolute neutrophil count (AUC: 0.816, *p* < 0.001), and absolute lymphocyte count (AUC: 0.804, *p* < 0.001) were good predictors of severe COVID‐19 among the participants. Other immune‐inflammatory markers were poorly associated with severe COVID‐19 in adults (Figure 1).

## 4 Discussion

The advent of COVID‐19 has changed public health landscapes worldwide, with its consequences principally felt in developing countries including Ghana, where healthcare resources are limited [1]. SARS‐CoV‐2 mainly colonizes the respiratory system; however, the virus interacts with other organs through ACE‐2 causing systemic damages and disrupting physiological processes including hemopoietic activities [4, 5, 6]. In this study, we profiled hematological abnormalities associated with SARS‐CoV‐2‐infected adults.

The COVID‐19‐infected patients included in this study were mostly young adults between 25 and 40 years, and had median age of 35.0 (29.0–47.0) years. This age group represents an energetic period in life where people actively engage in several activities, work assiduously to meet the needs of themselves and their associates. This adventure increases chances of such youthful adults of being exposed to SARS‐CoV‐2 as the virus is transmitted through respiratory droplets and aerosols during close contact with infected persons. The observation of high risk of young adults' exposure to COVID‐19 observed in this study is similar to findings from previous studies [29, 30, 31]. Ghanaian women interact more at well‐populated places such as market areas, funeral grounds and other social gatherings than men. This makes females a social conduit to facilitate the communication of SARS‐CoV‐2 among themselves, and accounts for the higher proportion of COVID‐19 detected among females than males in this study, as has been observed in other jurisdictions [8, 24, 32]. Other studies posit that males are more susceptible to COVID‐19 and its related severity and deaths due to the existence of excessive amount of ACE‐2 and transmembrane serine protease‐2 which facilitate disease progression [33, 34]. This study reports a higher proportion of SARS‐CoV‐2 infection among inhabitants living in urban settings than those in rural or peri‐urban areas, and this agrees with earlier concept [35]. Environmental factors such as high population density, crowded living and public spaces, greater mobility and travel, increased person‐to‐person contact, and higher exposure in congested hospitals may account for the observation of higher occurrence of COVID‐19 in urban settings.

Fever, cough, fatigue, headache, nausea, and loss of smell and taste were frequently reported symptoms among COVID‐19‐infected adults in this study. These findings corroborate other observations from earlier studies [4, 5, 33], and this highlights the diverse clinical manifestations of the disease. The presence of fever in 59.8% of patients coupled with the relative increase in body temperature in patients with severe COVID‐19 emphasizes the hyperinflammatory nature of SARS‐CoV‐2 infection, with the systemic release of inflammatory cytokines such as interferon gamma, IL‐1, IL‐6, and TNF‐α contributing extensively to fever pathogenesis [9, 31, 33]. Severe COVID‐19 patients experienced high systolic blood pressure and pulse rate, but lower oxygen saturation than adults with mild‐to‐moderate infection in this study. The decreased SpO_2_ could arise from the related enhanced lung damage and respiratory failure‐related hypoxia, whereas the direct effects of SARS‐CoV‐2 on renin‐angiotensin‐aldosterone system explains the elevated blood pressure in the patients [36, 37].

Anemia was the most common blood cell abnormality detected among the SARS‐CoV‐2 patients in this study, occurring in 60.4% of the patients. The anemia ranged from mild to severe, with a greater burden found among adults with severe COVID‐19 infection. The magnitude of anemia observed in SARS‐CoV‐2‐infected adults indicates the systemic involvement of the virus, which potentially suppresses erythropoiesis either directly via bone marrow suppression or indirectly through impaired nutrients metabolism, chronic inflammation, and renal dysfunction. Similar findings have been reported in previous studies in Ghana [6, 11, 12], and other settings [7, 8, 9]. The substantial relationship between anemia and adverse complications in COVID‐19 infection, including increased hospitalization, oxygen need, and mortality is reported in both developing and high‐income countries [10]. The prevalence of anemia was disproportionally higher in females than in males with COVID‐19, and this may be due to the positive androgenic effects on erythropoiesis which favors males and the periodic menstrual flow of females especially for those who had borderline hemoglobin prior to exposure to SARS‐CoV‐2 [8, 38]. Severe anemia was more common among the older (68.8%) and middle‐aged (30.8%) adults than the adolescents and the young adults with COVID‐19, which is similar to earlier reports [38, 39]. Also, older adults with COVID‐19 were 6.600 times, and middle‐aged adults were 4.435 times more likely to experience severe anemia than the adolescents. The higher susceptibility of the aged to anemia could be explained by the fact that with ageing only few bone marrows are hemopoietically active and more than half of such active bone marrows are occupied by fat, coupled with a significant drop of androgen especially in males after four decades in life and this has negative effect on blood cell production. Other probable etiology of anemia at older age could arise from bone marrow failure syndromes, presence of chronic kidney disease, and nutritional deficits [38, 40]. Similarly, COVID‐19 adults who resided in rural settings were 3.759 times higher than those living in the urban areas to experience anemia. This may be attributed to pre‐existing comorbidities and reduced access to healthcare. This finding suggests the need for targeted screening and intervention measures for susceptible populations, particularly in areas with limited diagnostic capacity.

The dominant forms of anemia in this study were normocytic normochromic and macrocytic normochromic; a distribution that reflects the multifactorial pathogenesis of COVID‐19‐associated anemia. Normocytic normochromic anemia is characteristically related to chronic inflammatory states, where cytokine‐triggered retardation of erythropoietin release and iron‐restricted erythropoiesis lead to reduced erythrocyte production but do not affect red cell sizes and hemoglobinization [39, 41]. On the contrary, macrocytosis may indicate impaired DNA synthesis in erythroblats, probably due to nutritional deficits, medications, or direct viral attack to the bone marrow [42, 43]. This spectrum of anemia forms highlight the relevance of a comprehensive diagnostic approach, mainly in settings where iron, folate, and B_12_ deficiencies are common.

In addition to anemia, a substantial proportion of COVID‐19 patients presented with thrombocytopenia (56.2%), largely of mild‐to‐moderate severity in this study. Thrombocytopenia in SARS‐CoV‐2 infection is multifactorial, involving direct immune‐mediated destruction of platelets, enhanced peripheral consumption in microthrombi, bone marrow suppression, and increased splenic sequestration [12, 13, 14, 15]. Platelet abnormalities, including microcytosis and altered platelet distribution width, were also found, suggesting compensatory marrow responses or disturbed megakaryopoiesis. These platelet abnormalities have been connected with poor outcomes in COVID‐19, and could serve as vital biomarkers for monitoring disease progression [13, 14]. Interestingly, while leucocytes count remained within normal for most COVID‐19 patients, specific leucocyte abnormalities were detected in this study. This study observed leucopenia and leucocytosis in 22.5% and 17.2% of the patients, respectively, demonstrating differing immune responses probably influenced by SARS‐CoV‐2 viral load, disease duration, and host factors. Neutropenia was observed in 40.8% of patients, while 39.6% demonstrated lymphocytosis among COVID‐19 adults. These findings are consistent with previous studies, where lymphocytosis was found in COVID‐19 patients with prolonged or severe infection, possibly indicating a delayed but overwhelmed adaptive immune response. Also, neutropenia, on the other hand, may occur through peripheral neutrophils consumption or bone marrow suppression due to the impact of inflammatory cytokines or direct SARS‐CoV‐2 invasion [16, 17, 18].

Systemic inflammatory ratios such as NLR, PLR, PNR, and SII were noticeably high in severe COVID‐19 patients in this study. These indices serve as surrogate markers of immune dysregulation and have been linked with worse clinical outcomes in various viral infections, including COVID‐19 [19, 20]. Although the predictive performance of these systemic inflammatory indices for severe COVID‐19 was poor in this study, this may be due to the population‐specific variances in immune response.

Surprisingly, only about one‐fourth of the COVID‐19 patients had normal blood cell parameters, and the rest exhibited either single‐cell abnormality, bicytopenia, or pancytopenia. The detection of bicytopenia (37.3%) and pancytopenia (17.8%) accentuates the systemic nature of COVID‐19 infection, which can affect multiple hemopoietic lineages. Pancytopenia has been associated with cytokine‐induced bone marrow dysfunction, viral tropism for hemopoietic progenitor cells, and consumptive coagulopathy—all of which can thwart clinical management and facilitate mortality risk [44].

### 4.1 Strength and Limitations

This study offers essential data on hematological abnormalities among adult COVID‐19 patients in Ghana. However, this study has some limitations. First, the cross‐sectional design restricts causal inference. Second, the study did not include healthy controls, preventing comparison with uninfected individuals. Third, the nutritional status of the participants was not evaluated. Lastly, participants were not screened for parasitic infections such as malaria and helminths. Regardless of these limitations, the study pinpoints vital hematological patterns that may support clinical management of COVID‐19.

## 5 Conclusion

Adults infected with SARS‐CoV‐2 experience diverse and clinically important hematological abnormalities. Anemia, thrombocytopenia, and leucocyte alterations are predominantly observed in COVID‐19, and often associate with disease severity. Increased inflammatory indices and multiple cytopenias are especially common in severely ill patients. Routine blood cell analysis is essential in the management of COVID‐19, particularly in resource‐limited settings. Future studies should investigate the longitudinal changes in hematological parameters and their relationship with COVID‐19 outcomes in different geographical areas and populations.

## Author Contributions

**Charles Nkansah:** conceptualization, investigation, writing – original draft, writing – review and editing, methodology, visualization, formal analysis, data curation, resources. **Felix Osei‐Boakye:** writing – original draft, writing – review and editing, visualization, formal analysis, methodology. **Samuel K. Appiah:** investigation, writing – original draft, writing – review and editing, methodology, data curation.

## Funding

The authors received no specific funding for this work.

## Conflicts of Interest

The authors declare no conflicts of interest.

## Supporting information

STROBE Statement—checklist of items that should be included in reports of observational studies.

## Data Availability

The data supporting findings in this study are available from the corresponding author upon reasonable request.

## References

[1] K. Dhama , S. Khan , R. Tiwari , et al., “Coronavirus Disease 2019‐COVID‐19,” Clinical Microbiology Reviews 33 (2020): e00028‐20, 10.1128/CMR.00028-20. 32580969 PMC7405836

[2] W.H.O . WHO COVID‐19 dashboard. 2025.

[3] E. Kenu , J. Frimpong , and K. Koram , “Responding to the COVID‐19 Pandemic in Ghana,” Ghana Medical Journal 54 (2020): 72–73, 10.4314/gmj.v54i2.1. 33536675 PMC7829051

[4] C. B. Beggs , R. Abid , F. Motallebi , A. Samad , N. Venkatesan , and E. J. Avital , “Airborne Transmission of SARS‐CoV‐2: The Contrast Between Indoors and Outdoors,” Fluids 9 (2024): 54, 10.3390/fluids9030054.

[5] P. Bhadoria and H. Rathore , “Multi‐Organ System Dysfunction in Covid‐19—A Review,” Journal of Evolution of Medical and Dental Sciences 10 (2021): 632–637, 10.14260/jemds/2021/135.

[6] E. B. Ackah , M. Owusu , B. Sackey , et al., “Hematological Profile and ACE2 Levels of COVID‐19 Patients in a Metropolis in Ghana,” COVID 4 (2024): 117–129, 10.3390/covid4020011.

[7] J. L. Benoit , S. W. Benoit , M. H. S. de Oliveira , G. Lippi , and B. M. Henry , “Anemia and COVID‐19: A Prospective Perspective,” Journal of Medical Virology 93 (2021): 708–711, 10.1002/jmv.26530. 32949170 PMC7536957

[8] G. Bergamaschi , F. Borrelli de Andreis , N. Aronico , et al., “Anemia in Patients With Covid‐19: Pathogenesis and Clinical Significance,” Clinical and Experimental Medicine 21 (2021): 239–246, 10.1007/s10238-020-00679-4. 33417082 PMC7790728

[9] L. Abu‐Ismail , M. J. J. Taha , M. T. Abuawwad , et al., “COVID‐19 and Anemia: What Do We Know So Far?,” Hemoglobin 47 (2023): 122–129, 10.1080/03630269.2023.2236546. 37519257

[10] J. Sheikh , H. Lawson , J. Allotey , et al., “Global Variations in the Burden of SARS‐CoV‐2 Infection and Its Outcomes in Pregnant Women by Geographical Region and Country's Income Status: A Meta‐Analysis,” BMJ Global Health 7 (2022): e010060, 10.1136/bmjgh-2022-010060. PMC9659713 36368768

[11] Y. Konlaan , S. Asamoah Sakyi , K. Kumi Asare , et al., “Evaluating Immunohaematological Profile Among COVID‐19 Active Infection and Recovered Patients in Ghana,” PLoS One 17 (2022): e0273969, 10.1371/journal.pone.0273969. 36094915 PMC9467340

[12] K. Mensah , V. O. Ofori , C. Nkansah , et al., “Blood Cell Indices and Morphological Abnormalities Detected Among COVID‐19 Patients Receiving Care,” SciMedicine Journal 5 (2023): 8–18, 10.28991/scimedj-2023-05-01-02.

[13] E. Gavriilaki , I. Sakellari , M. Gavriilaki , and A. Anagnostopoulos , “Thrombocytopenia in COVID‐19: Pathophysiology Matters,” Annals of Hematology 100 (2021): 2139–2140, 10.1007/s00277-020-04183-3. 32683455 PMC7368623

[14] T. Iba and J. H. Levy , “Thrombosis and Thrombocytopenia in COVID‐19 and After COVID‐19 Vaccination,” Trends in Cardiovascular Medicine 32 (2022): 249–256, 10.1016/j.tcm.2022.02.008. 35202800 PMC8861143

[15] T. Patel , N. Stanton , I. Gkikas , and D. I. D. Triantafyllopoulou , “Severe Thrombocytopaenia Secondary to COVID‐19,” BMJ Case Reports 13 (2020): e237645, 10.1136/bcr-2020-237645. PMC7493096 32933915

[16] X. Tong , A. Cheng , X. Yuan , et al., “Characteristics of Peripheral White Blood Cells in COVID‐19 Patients Revealed by a Retrospective Cohort Study,” BMC Infectious Diseases 21 (2021): 1236, 10.1186/s12879-021-06899-7. 34886793 PMC8655490

[17] B. Zhu , X. Feng , C. Jiang , et al., “Correlation Between White Blood Cell Count at Admission and Mortality in COVID‐19 Patients: A Retrospective Study,” BMC Infectious Diseases 21 (2021): 574, 10.1186/s12879-021-06277-3. 34126954 PMC8202964

[18] A. Anurag , P. K. Jha , and A. Kumar , “Differential White Blood Cell Count in the COVID‐19: A Cross‐Sectional Study of 148 Patients,” Diabetes & Metabolic Syndrome: Clinical Research & Reviews 14 (2020): 2099–2102, 10.1016/j.dsx.2020.10.029. PMC7605785 33160224

[19] S. Gopalakrishnan , B. Krishnan , M. S. Krishnan , S. Kandasamy , P. M. Sahul Hameed , and V. Karunakaran , “The Prognostic Role of Inflammatory Markers in COVID‑19 Patients: A Retrospective Analysis in a Tertiary Care Hospital of Southern India,” Journal of Current Research in Scientific Medicine 8 (2022): 108–115, 10.4103/jcrsm.jcrsm_4_22.

[20] R. Suvarna , M. Biswas , V. Shenoy Belle , and V. Shanbhag , “Clinical Features and Risk Predictor Markers in COVID‐19 Patients in Correlation With Disease Severity and Comorbidities—A Data‐Based Retrospective Study,” YMER Digital 21 (2022): 160–184, 10.37896/ymer21.04/15.

[21] J. W. Kosidło , B. Wolszczak‐Biedrzycka , J. Matowicka‐Karna , V. Dymicka‐Piekarska , and J. Dorf , “Clinical Significance and Diagnostic Utility of NLR, LMR, PLR and SII in the Course of COVID‐19: A Literature Review,” Journal of Inflammation Research 16 (2023): 539–562, 10.2147/JIR.S395331. 36818192 PMC9930576

[22] W. Xia , Y. Tan , S. Hu , C. Li , and T. Jiang , “Predictive Value of Systemic Immune‐Inflammation Index and Neutrophil‐To‐Lymphocyte Ratio in Patients With Severe COVID‐19,” Clinical and Applied Thrombosis/Hemostasis 28 (2022): 1–7, 10.1177/10760296221111391. PMC9247370 35765218

[23] A. G. Fois , P. Paliogiannis , V. Scano , et al., “The Systemic Inflammation Index on Admission Predicts In‐Hospital Mortality in COVID‐19 Patients,” Molecules 25 (2020): 5725, 10.3390/molecules25235725. 33291581 PMC7731255

[24] M. Owusu , A. A. Sylverken , S. T. Ankrah , et al., “Epidemiological Profile of SARS‐CoV‐2 Among Selected Regions in Ghana: A Cross‐Sectional Retrospective Study,” PLoS One 15 (2020): e0243711, 10.1371/journal.pone.0243711. 33301533 PMC7728229

[25] F. Osei‐Boakye , C. Nkansah , S. K. Appiah , et al., “Seroprevalence, Trends, and Risk Factors of Hepatitis B and C Among Family Replacement Blood Donors; A 7‐Year Retrospective Study at Sunyani Municipal Hospital, Ghana,” Journal of Immunoassay and Immunochemistry 44 (2023): 162–175, 10.1080/15321819.2023.2168555. 36656031

[26] W.H.O . “Clinical Management of COVID‐19: Living Guideline—September 15, 2022. 2025, http://apps.who.int/bookorders.%0Ahttps://apps.who.int/iris/bitstream/handle/10665/362783/WHO-2019-nCoV-Clinical-2022.2-eng.pdf?sequence=1&isAllowed=y%0Ahttps://fi-admin.bvsalud.org/document/view/85mxm. 35917394

[27] A. Aggarwal , A. Aggarwal , S. Goyal , and S. Aggarwal , “Iron‐Deficiency Anemia Among Adolescents: A Global Public Health Concern,” International Journal of Advanced Community Medicine 3 (2020): 35–40, 10.33545/comed.2020.v3.i2a.148.

[28] D. Sakzabre , E. A. Asiamah , E. E. Akorsu , et al., “Haematological Profile of Adults With Malaria Parasitaemia Visiting the Volta Regional Hospital, Ghana,” Advances in Hematology 2020 (2020): 1–6, 10.1155/2020/9369758. PMC7036090 32095139

[29] M. Manivannan , M. P. Jogalekar , M. S. Kavitha , B. A. V. Maran , and P. Gangadaran , “A Mini‐Review on the Effects of COVID‐19 on Younger Individuals,” Experimental Biology and Medicine 246 (2021): 293–297, 10.1177/1535370220975118. 33210552 PMC7859671

[30] R. I. Silliman Cohen and E. A. Bosk , “Vulnerable Youth and the COVID‐19 Pandemic,” Pediatrics 146 (2020): e20201306, 10.1542/peds.2020-1306. 32345686

[31] C. Nkansah , M. Owusu , S. K. Appiah , et al., “Effects of COVID‐19 Disease on PAI‐1 Antigen and Hematological Parameters During Disease Management: A Prospective Cross‐Sectional Study in a Regional Hospital in Ghana,” PLOS Global Public Health 3 (2023): 1–19, 10.1371/journal.pgph.0001866. PMC10286987 37347738

[32] A. Krueger , J. K. L. Gunn , J. Watson , et al., “Characteristics and Outcomes of Contacts of COVID‐19 Patients Monitored Using an Automated Symptom Monitoring Tool—Maine, May–June 2020,” MMWR. Morbidity and Mortality Weekly Report 69 (2020): 1026–1030, https://pubmed.ncbi.nlm.nih.gov/32759918/. 32759918 10.15585/mmwr.mm6931e2 PMC7454893

[33] J. R. Patel , B. C. Amick , K. S. Vyas , E. Bircan , D. Boothe , and W. N. Nembhard , “Gender Disparities in Symptomology of COVID‐19 Among Adults in Arkansas,” Preventive Medicine Reports 35 (2023): 102290, 10.1016/j.pmedr.2023.102290. 37441188 PMC10289819

[34] Y. J. Su , K. C. Kuo , T. W. Wang , and C. W. Chang , “Gender‐Based Differences in COVID‐19,” New Microbes and New Infections 42 (2021): 100905, 10.1016/j.nmni.2021.100905. 34031638 PMC8133826

[35] D. F. Cuadros , A. J. Branscum , Z. Mukandavire , F. D. Miller , and N. MacKinnon , “Dynamics of the COVID‐19 Epidemic in Urban and Rural Areas in the United States,” Annals of Epidemiology 59 (2021): 16–20, 10.1016/j.annepidem.2021.04.007. 33894385 PMC8061094

[36] M. Vicenzi , R. Di Cosola , M. Ruscica , et al., “The Liaison Between Respiratory Failure and High Blood Pressure: Evidence From COVID‐19 Patients,” European Respiratory Journal 56 (2020): 2001157, 10.1183/13993003.01157-2020. 32430432 PMC7241109

[37] S. Chatterjee , T. Sengupta , S. Majumder , and R. Majumder , “Place Time and Date No of Patient Sex Age Mortality Male Female Netherlands[42],” Aging (Albany NY) 12 (2020): 15954–15961, 10.18632/aging.103869. 32826388 PMC7485709

[38] P. E. Taneri , S. A. Gómez‐Ochoa , E. Llanaj , et al., “Anemia and Iron Metabolism in COVID‐19: A Systematic Review and Meta‐Analysis,” European Journal of Epidemiology 35 (2020): 763–773, 10.1007/s10654-020-00678-5. 32816244 PMC7438401

[39] V. Gürsoy , S. Avci , S. Ermurat , A. Erol , and M. Yazici , “Anemia and COVID‐19,” European Research Journal 9 (2023): 1074–1082, 10.18621/eurj.1169438.

[40] R. Stauder , P. Valent , and I. Theurl , “Anemia at Older Age: Etiologies, Clinical Implications, and Management,” Blood 131 (2018): 505–514, 10.1182/blood-2017-07-746446. 29141943

[41] A. Gupta , “Decision Making Through Problem Based Learning in Hematology,” in Decision Making Through Problem Based Learning in Hematology (Springer, 2024), 63–75, 10.1007/978-981-99-8933-1.

[42] V. G. Soylu , S. Gülten , A. Yilmaz , Ö. Taşkın , U. Demir , and F. Çatan , “Is Macrocytic Erythrocyte a New Prognostic Parameter in Critical COVID‐19 Disease?,” Journal of Health Science and Medical Research 4 (2021): 828–834, 10.32322/jhsm.971934.

[43] I. H. Yasak , M. Yilmaz , E. S. Seyhanli , and A. Gonel , “Relationship Between Coronavirus Disease and Erythrocyte Morphology Parameters,” International Journal of Current Medical and Biological Sciences 2 (2022): 192–198, 10.5281/zenodo.7272861Highlights.

[44] M. Raadsen , J. du Toit , T. Langerak , B. van Bussel , E. van Gorp , and M. Goeijenbier , “Thrombocytopenia in Virus Infections,” Journal of Clinical Medicine 10 (2021): 877, 10.3390/jcm10040877. 33672766 PMC7924611

